# Supplementary material for: Sam68 promotes hepatic gluconeogenesis via CRTC2
Source: Nat Commun. 2021 Jun 7;12:3340. doi: 10.1038/s41467-021-23624-9 (PMC8185084; doi:10.1038/s41467-021-23624-9)
Supplement: Supplementary file 1 — Supplementary Information [file 41467_2021_23624_MOESM1_ESM.pdf]

Sam68 promotes hepatic gluconeogenesis via CRTC2

Aijun Qiao[1], Junlan Zhou[2], Shiyue Xu[1], Wenxia Ma[1], Chan Boriboun[1], Teayoun Kim[3], Baolong Yan[1], Jianxin Deng[1], Liu Yang[1], Eric Zhang[1], Yuhua Song[1], Yongchao C. Ma[4], Stephane Richard[5], Chunxiang Zhang[1], Hongyu Qiu[6], Kirk M Habegger[3], Jianyi Zhang[1], Gangjian Qin[1,2*]

[1] Department of Biomedical Engineering, University of Alabama at Birmingham, School of Medicine and School of Engineering, Birmingham, AL 35294, USA
[2] Feinberg Cardiovascular Research Institute, Northwestern University Feinberg School of Medicine, Chicago, IL 60611, USA
[3] Department of Medicine - Endocrinology, Diabetes & Metabolism, University of Alabama at Birmingham, School of Medicine, Birmingham, AL 35294, USA
[4] Departments of Pediatrics, Neurology and Physiology, Northwestern University Feinberg School of Medicine, Anne & Robert H. Lurie Children's Hospital of Chicago, Chicago, IL, 60611, USA
[5] Lady Davis Institute for Medical Research, McGill University, Montreal, Quebec, Canada
[6] Center of Molecular and Translational Medicine, Institution of Biomedical Science, Georgia State University, Atlanta, GA 30303, USA

Supplementary Information

Supplementary Figures

Figure S1

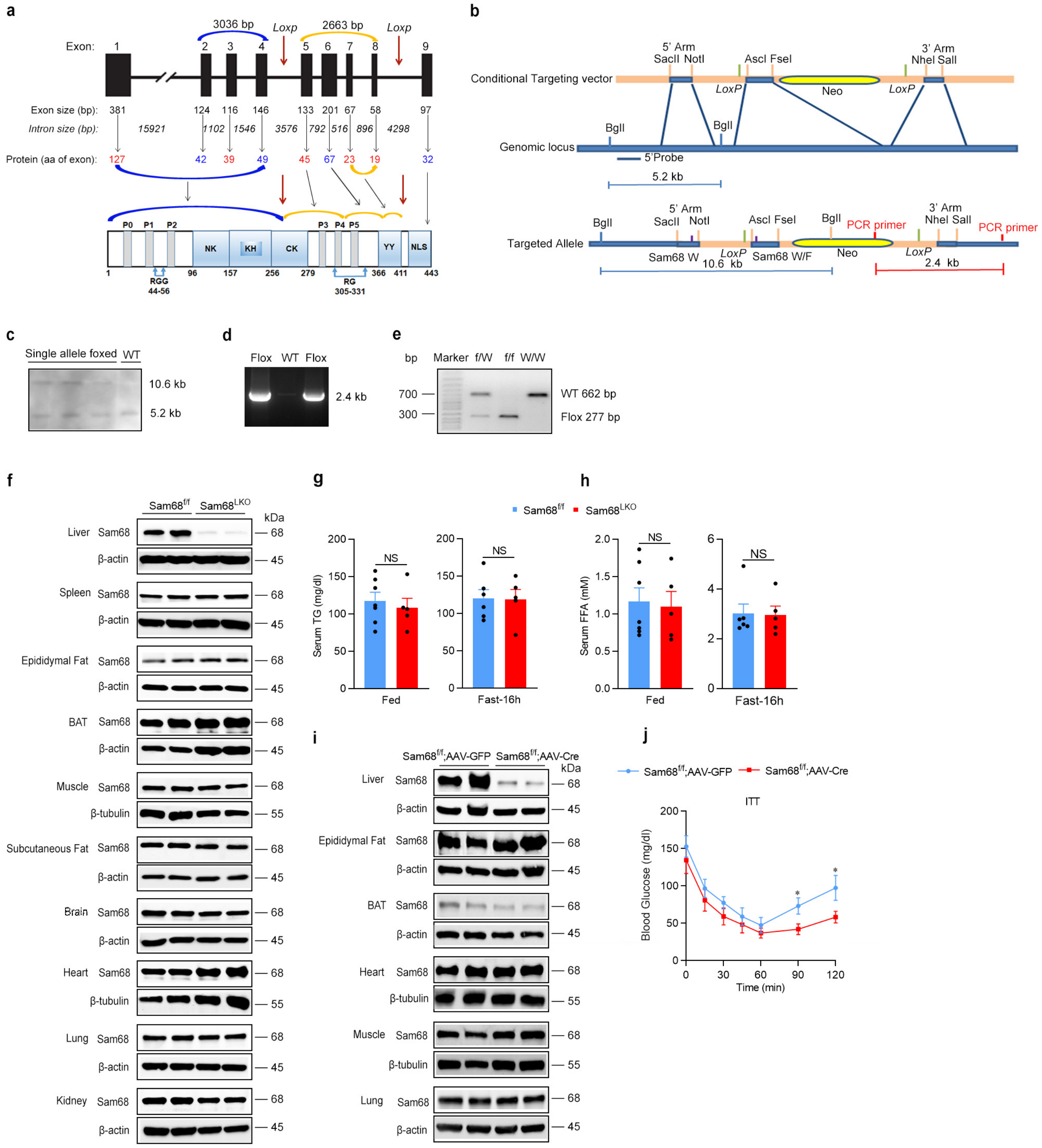
a
b
c
d
e
f
g
h
i
j
Exon:
Exon size (bp):
Intron size (bp):
Protein (aa of exon):
3036 bp
2663 bp
Loxp
Conditional Targeting vector
Genomic locus
Targeted Allele
5' Arm
3' Arm
Neo
LoxP
5'Probe
5.2 kb
10.6 kb
2.4 kb
PCR primer
Single allele foxed
WT
Flox
Marker
WT 662 bp
Flox 277 bp
Sam68
β-actin
β-tubulin
kDa
Liver
Spleen
Epididymal Fat
BAT
Muscle
Subcutaneous Fat
Brain
Heart
Lung
Kidney
Serum TG (mg/dl)
Serum FFA (mM)
NS
Fed
Fast-16h
ITT
Blood Glucose (mg/dl)
Time (min)

Supplementary Fig. 1: Generation and metabolic characterization of hepatocyte-specific *Sam68* knockout mice. (**a**) The structural components of the *Sam68* gene (*upper* panel*)* and protein (*lower* panel) are illustrated. Sam68 contains a GSG domain composed of a single RNA-binding KH domain flanked by NK (N-terminus of KH) and CK (C-terminus of KH) segments; six consensus proline-rich motifs (P0-P5); RGG boxes; a C-terminal tyrosine-rich domain (YY), and a nuclear localization signal (NLS). Amino acid positions are numbered across the bottom of the protein structure. (**b**) The gene-targeting strategy for floxing exons 5-8 of *Sam68* in embryonic stem (ES) cells is illustrated. (**c**) Southern blotting and (**d**) PCR analyses were conducted to confirm that the ES clones were correctly targeted. (**e**) The genotypes of the WT and floxed alleles were confirmed via PCR. (**f**) Sam68 protein expression was evaluated via Western blot in the liver, spleen, epididymal fat, brown fat, skeletal muscle, subcutaneous fat, brain, heart, lung, and kidney tissues of $Sam68^{LKO}$ and $Sam68^{f/f}$ mice. (**g**) Serum triglyceride and (**h**) free fatty acid levels were measured in $Sam68^{LKO}$ and $Sam68^{f/f}$ mice under feeding ($Sam68^{f/f}$, n=7; $Sam68^{LKO}$, n=5) or 16-h fasting condition ($Sam68^{f/f}$, n=6; $Sam68^{LKO}$, n=5). (**i**) $Sam68^{f/f}$ mice were injected with AAV8-TBG-iCre ($Sam68^{f/f}$;AAV-Cre) to induce a hepatic-specific *Sam68* deletion or with control AAV8-TBG-eGFP ($Sam68^{f/f}$;AAV-GFP). Three weeks later, Sam68 protein levels were evaluated via Western blot in the liver, epididymal fat, brown fat, heart, skeletal muscle, and lung tissues. (**j**) Blood glucose levels were measured in $Sam68^{f/f}$;AAV-Cre (n=8) and $Sam68^{f/f}$;AAV-GFP (n=8) mice at the indicated time points during the ITT. Data are expressed as mean ± standard error of the mean (s.e.m.). *$p<0.05$ (g-h, unpaired two-sided *t* test; j, two-way ANOVA). Source data are provided as a Source Data file.

Figure S2

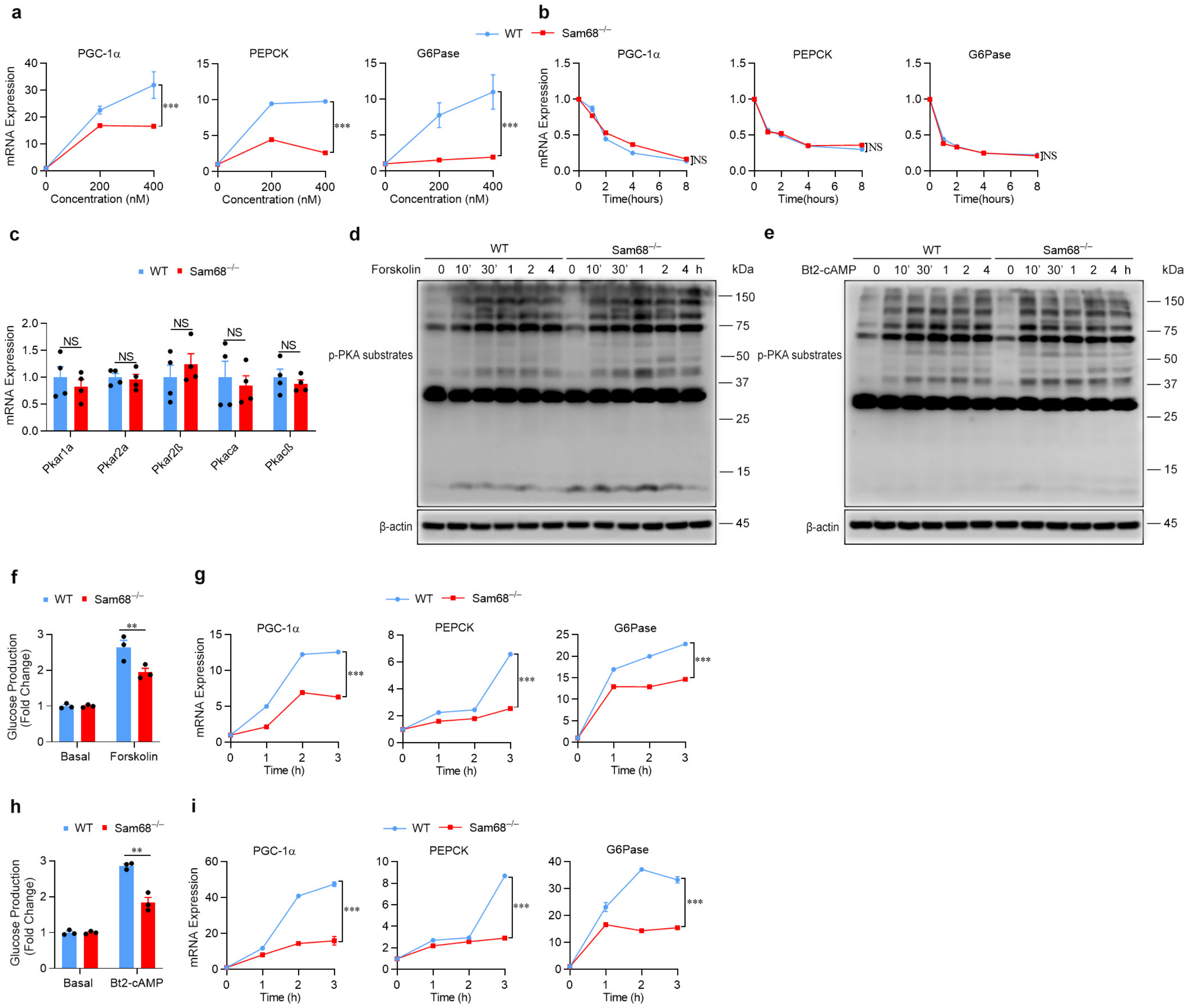

a
b
WT
Sam68−/−
PGC-1α
PEPCK
G6Pase
mRNA Expression
Concentration (nM)
Time(hours)
***
NS
c
Pkar1a
Pkar2a
Pkar2ß
Pkaca
Pkacß
d
e
Forskolin
Bt2-cAMP
0 10' 30' 1 2 4 h
kDa
p-PKA substrates
β-actin
150
75
50
37
25
15
45
f
g
h
i
Glucose Production (Fold Change)
Basal
Forskolin
Bt2-cAMP
**
Time (h)

Supplementary Fig. 2: *Sam68* deficiency reduces glucagon signaling and glucose production in hepatocytes. (**a**) WT and *Sam68*$^{-/-}$ hepatocytes were treated with 200 nM and 400 nM glucagon for 3 h (n=3), and then the mRNA expression of gluconeogenic genes was measured via qRT-PCR. (**b**) WT and *Sam68*$^{-/-}$ hepatocytes were treated with glucagon (100 nM) for 3 h and then with Actinomycin D (10 μg/mL) to block new mRNA synthesis; mRNA expression of gluconeogenic genes was measured 0-8 h later (n=3). (**c**) mRNA expression of PKA subunits was measured in WT and *Sam68*$^{-/-}$ hepatocytes (n=4). (**d-e**) WT and *Sam68*$^{-/-}$ primary hepatocytes were treated with (**d**) forskolin (10 μM) or (**e**) Bt2-cAMP (100 μM) for 0-30 min and 1-4 h; then, protein levels of phosphorylated PKA substrates was evaluated via Western blot. (**f**) Glucose production was measured in WT and *Sam68*$^{-/-}$ primary hepatocytes after treatment with forskolin for 4 h (n=3). (**g**) mRNA expression of gluconeogenic genes was measured in WT and Sam68$^{-/-}$ hepatocytes after treatment with forskolin for 0-3 h (n=3). (**h**) Glucose production was measured in WT and *Sam68*$^{-/-}$ primary hepatocytes after treatment with Bt2-cAMP for 4 h (n=3). (**i**) mRNA expression of gluconeogenic genes was measured in WT and *Sam68*$^{-/-}$ hepatocytes after treatment with Bt2-cAMP for 0-3 h (n=3). Data are expressed as mean ± standard error of the mean (s.e.m.). *p<0.05, **p<0.01, ***p<0.001, NS, not significant (a-b, f-i: two-way ANOVA; c: unpaired two-sided *t* test). “n” denotes biologically independent primary hepatocyte samples. Source data are provided as a Source Data file.

Figure S3

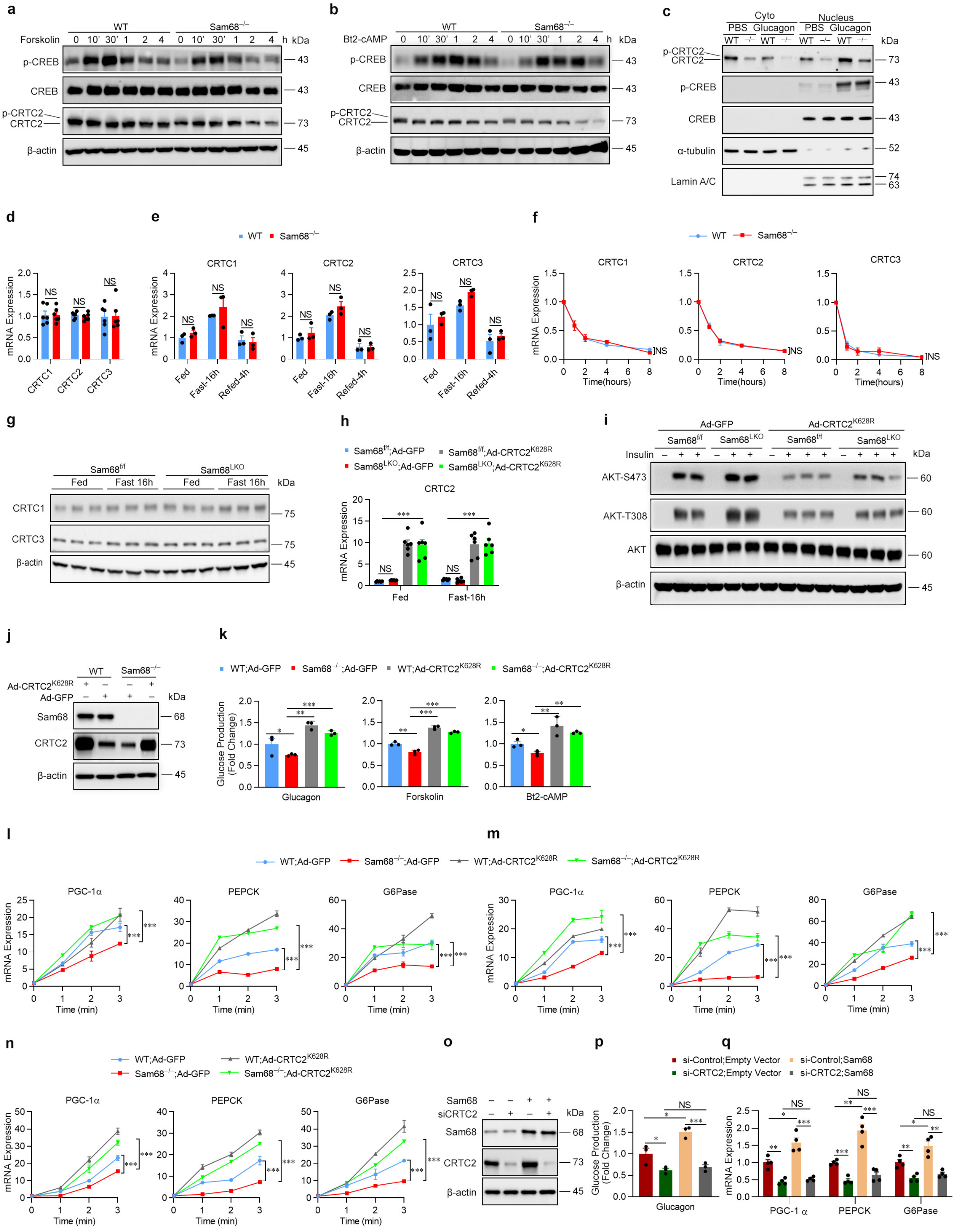

Supplementary Fig. 3: Hepatic *Sam68* deficiency reduces glucagon signaling and CRTC2 protein stability. (**a-b**) WT and *Sam68*$^{-/-}$ primary hepatocytes were treated with (**a**) forskolin (10 μM) and (**b**) Bt2-cAMP(100 μM) for 0-30 min and 1-4 h; then, protein levels of phosphorylated CREB (p-CREB), total CREB, and CRTC2 were evaluated via Western blot. (**c**) WT and *Sam68*$^{-/-}$ primary hepatocytes were treated with PBS or glucagon (100 nM) for 30 min; then, protein levels of p-CREB, total CREB, and CRTC2 in nuclear and cytoplasmic extracts were evaluated by immunoblot. (**d-e**) mRNA expression of *CRTC1*, *CRTC2*, and *CRTC3* was evaluated via qRT-PCR in (**d**) WT and *Sam68*$^{-/-}$ primary hepatocytes (n=6) and in (**e**) liver tissue from WT and *Sam68*$^{-/-}$ mice (n=3) under feeding conditions, after 16 h of fasting, and after 16 h of fasting followed by 4 h of refeeding. (**f**) mRNA expression of *CRTC1*, *CRTC2*, and *CRTC3* was evaluated in WT and *Sam68*$^{-/-}$ hepatocytes after treatment with Actinomycin D (10 μg/mL) for 0-8h to block new mRNA synthesis (n=3). (**g**) Protein expression of CRTC1 and CRTC3 was evaluated by Western blotting in the liver of *Sam68*^{LKO} and *Sam68*$^{f/f}$ mice under feeding condition or after 16 h fasting. (**h-i**) *Sam68*^{LKO} and *Sam68*$^{f/f}$ mice were administered Ad-GFP or Ad-CRTC2^{K628R}. (**h**) Four days later, mice were sacrificed under feeding conditions or after 16 h of fasting, and *CRTC2* mRNA expression was evaluated in liver tissues (n=6). (**i**) Four days after Ad-GFP or Ad-CRTC2^{K628R} administration, mice were treated with (+) or without (–) insulin (1 mU/g), and protein levels of phosphorylated AKT (at amino acids S473 and T308) and total AKT were evaluated 20 min later. (**j-n**) WT and *Sam68*$^{-/-}$ primary hepatocytes were treated with Ad-GFP or mutant Ad-CRTC2^{K628R}; 48 h later, (**j**) CRTC2 protein expression was evaluated via Western blot, (**k**) glucose production was measured after treatment with glucagon (100 nM), forskolin (10 μM) or Bt2-cAMP (100 μM) for 4 h, and (**l-n**) mRNA expression of gluconeogenic genes was evaluated after treatment with (**l**) glucagon, (**m**) forskolin, or (**n**) Bt2-cAMP for 0-3 h (n=3). (**o-q**) HepG2 cells were co-transfected with a Sam68-expressing or empty plasmid and a CRTC2 or scrambled siRNA for 48 h, then treated with glucagon for 4 h, followed by assessments of (**o**) Sam68 and CRTC2 protein expression, (**p**) glucose production (n=3), and (**q**) gluconeogenesis gene expression (n=4). Data are expressed as mean ± standard error of the mean (s.e.m.). *p<0.05, **p<0.01, ***p<0.001, NS, not significant (d: unpaired two-sided *t* test; e-f, h, k-n, p-q: two-way ANOVA). “n” denotes biologically independent primary hepatocyte samples or liver tissues or independently transfected HepG2 cell samples. Source data are provided as a Source Data file.

Figure S4

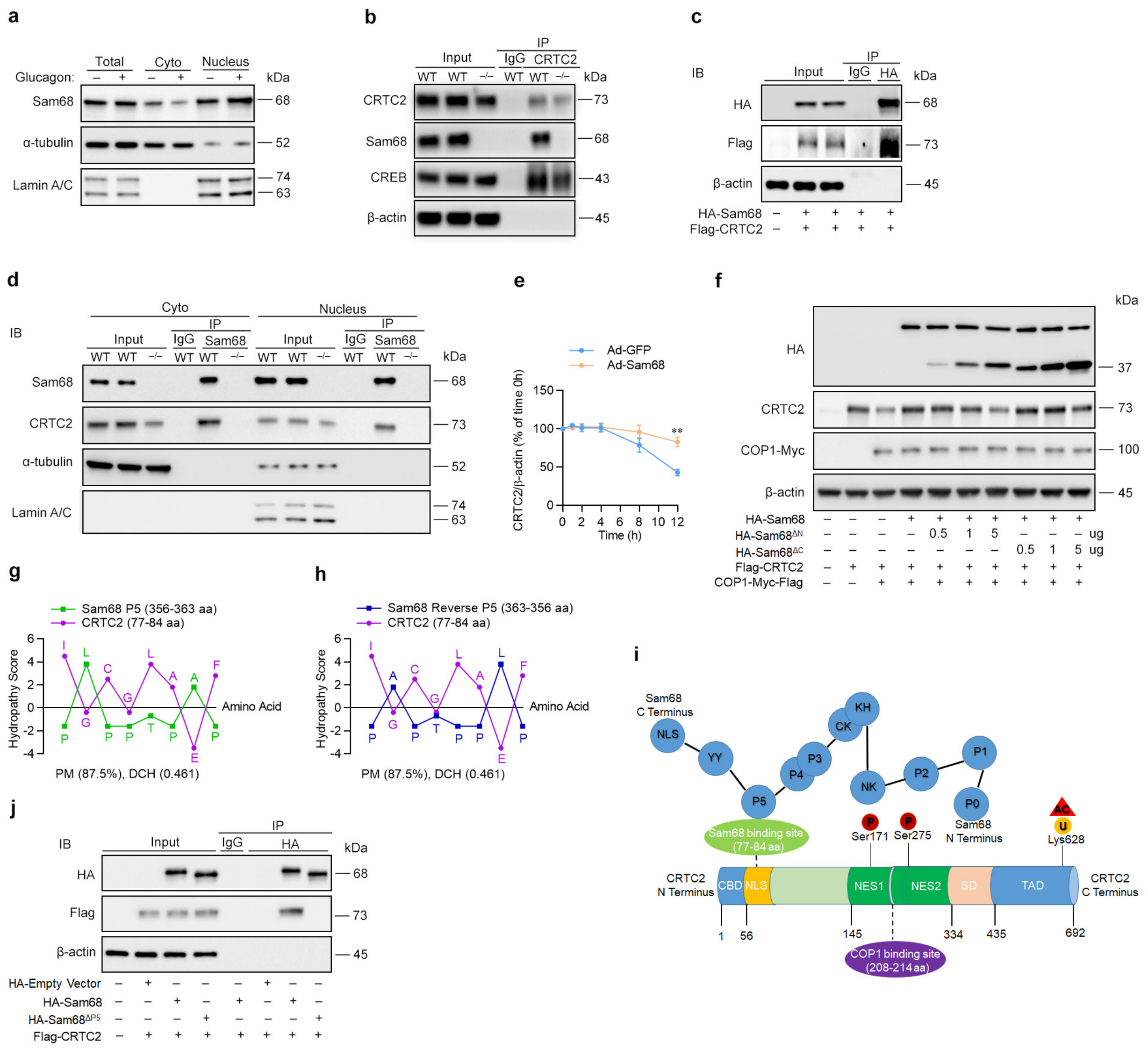
a
Total
Cyto
Nucleus
Glucagon:
kDa
Sam68
α-tubulin
Lamin A/C
b
IP
Input
IgG
CRTC2
CRTC2
Sam68
CREB
β-actin
c
IB
Input
IgG
HA
HA
Flag
β-actin
HA-Sam68
Flag-CRTC2
d
Cyto
Nucleus
e
Ad-GFP
Ad-Sam68
CRTC2/β-actin (% of time 0h)
Time (h)
f
COP1-Myc
COP1-Myc-Flag
g
Sam68 P5 (356-363 aa)
CRTC2 (77-84 aa)
Hydropathy Score
Amino Acid
PM (87.5%), DCH (0.461)
h
Sam68 Reverse P5 (363-356 aa)
i
Sam68 C Terminus
Sam68 binding site (77-84 aa)
Ser171
Ser275
Sam68 N Terminus
Lys628
CRTC2 N Terminus
CRTC2 C Terminus
CBD
NLS
NES1
NES2
SD
TAD
COP1 binding site (208-214 aa)
j
HA-Empty Vector
HA-Sam68
Flag-CRTC2

Supplementary Fig. 4: Sam68 interacts directly with CRTC2. (**a**) WT primary hepatocytes were treated with glucagon (100 nM) or PBS for 30 min, then, Sam68 protein levels were evaluated in nuclear and cytoplasmic fractions by immunoblot. (**b**) WT and *Sam68*$^{-/-}$ primary hepatocytes were treated with glucagon (100 nM) for 30 min; then, CRTC2 was immunoprecipitated, and Sam68 was detected in the precipitate by immunoblot. (**c**) 293T cells were co-transfected with plasmids coding for HA-tagged Sam68 and Flag-tagged CRTC2; 48 h later, Sam68 was immunoprecipitated from the cells with anti-HA, and CRTC2 was detected in the precipitates by immunoblotting with anti-Flag. (**d**) Nuclear and cytoplasm protein extracts were isolated from WT and *Sam68*$^{-/-}$ primary hepatocytes; Sam68 was immunoprecipitated, and CRTC2 in the precipitate of each fraction was detected by immunoblot. (**e**) WT primary hepatocytes were transduced with Ad-GFP or Ad-Sam68, treated by cycloheximide (100 μM) for 0-12 h; then CRTC2 protein levels were evaluated by Western blot, quantified via NIH image J software, normalized to β-actin levels, and reported as the proportion of the amount present at 0 h. Data are expressed as mean ± standard error of the mean (s.e.m.). **p<0.001 (two-way ANOVA). (**f**) HepG2 cells were co-transfected with three plasmids, one coding for Flag-tagged CRTC2, one for Myc-Flag-tagged COP1, and one for HA-tagged WT Sam68 or for Sam68$^{\Delta N}$ or Sam68$^{\Delta C}$ mutant; 48 h later, protein levels of CRTC2, COP1, and Sam68 or Sam68$^{\Delta N}$ or Sam68$^{\Delta C}$ were evaluated via immunoblotting with anti-CRTC2, anti-Myc, and anti-HA antibodies, respectively. (**g-i**) Computational results from text pattern search and hydropathy analyses show that P5 domain in the C-terminus of Sam68 has 87.5% hydropathic complementarity/percent match (PM) and 0.461 degree of complementary hydropathy (DCH) with the N-terminus of CRTC2 (amino acids 77-84) in a palindromic manner (**g** and **h**), suggesting a model that P5 domain in Sam68 binds N-terminal nuclear localization domain of CRTC2 (amino acids 77-84) (**i**). (**j**) 293T cells were co-transfected with two plasmids, one coding for Flag-tagged CRTC2 and one for HA-tagged WT Sam68, HA-tagged Sam68$^{\Delta P5}$, or HA only; 48 h later, HA was immunoprecipitated from the cells, and CRTC2 in the precipitates was detected by immunoblotting with anti-Flag. Source data are provided as a Source Data file.

Figure S5

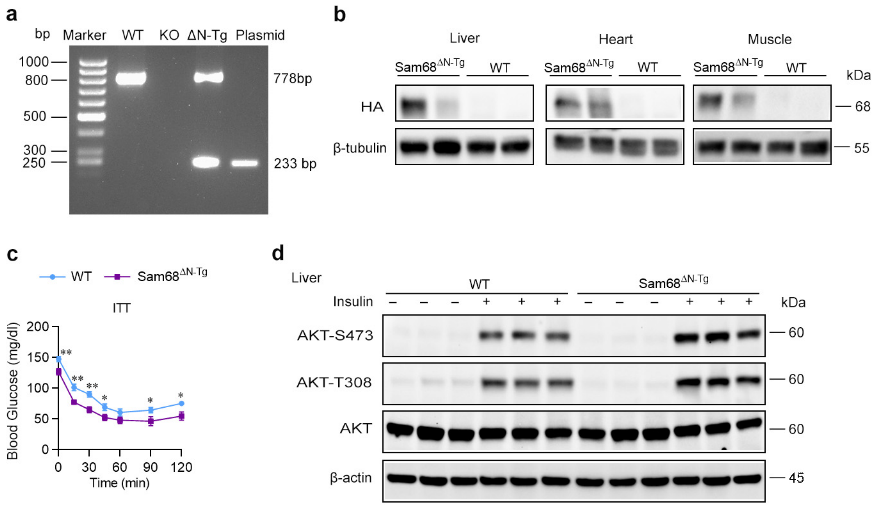
a
bp
Marker
WT
KO
ΔN-Tg
Plasmid
1000
800
500
300
250
778bp
233 bp
b
Liver
Heart
Muscle
Sam68ΔN-Tg
WT
kDa
HA
β-tubulin
68
55
c
WT
Sam68ΔN-Tg
ITT
Blood Glucose (mg/dl)
Time (min)
d
Liver
Insulin
AKT-S473
AKT-T308
AKT
β-actin
60
45

Supplementary Fig. 5: Generation and metabolic characterization of *Sam68*$^{\Delta N\text{-}Tg}$ mice. Experiments were conducted in mice carrying an HA-tagged ΔN-truncated Sam68 mutation (*Sam68*$^{\Delta N\text{-}Tg}$) and their WT littermates. (**a**) The genotypes of WT and *Sam68*$^{\Delta N\text{-}Tg}$ mice were confirmed via PCR. (**b**) Expression of the Sam68$^{\Delta N}$ mutant protein was evaluated in the liver, heart, and skeletal muscle tissues of *Sam68*$^{\Delta N\text{-}Tg}$ and WT mice via immunoblot with anti-HA. (**c**) Blood glucose levels during the insulin tolerance test (ITT) were measured in *Sam68*$^{\Delta N\text{-}Tg}$ (n=9) and WT (n=11) mice. (**d**) *Sam68*$^{\Delta N\text{-}Tg}$ and WT mice were treated with (+) or without (–) insulin (1 U/kg); 20 minutes later, liver tissues were harvested and protein levels of phosphorylated AKT (at amino acids S473 and T308) and total AKT were evaluated by Western blot (n=3). Data are expressed as mean ± standard error of the mean (s.e.m.). *p<0.05, **p<0.01 (two-way ANOVA). Source data are provided as a Source Data file.

Figure S6

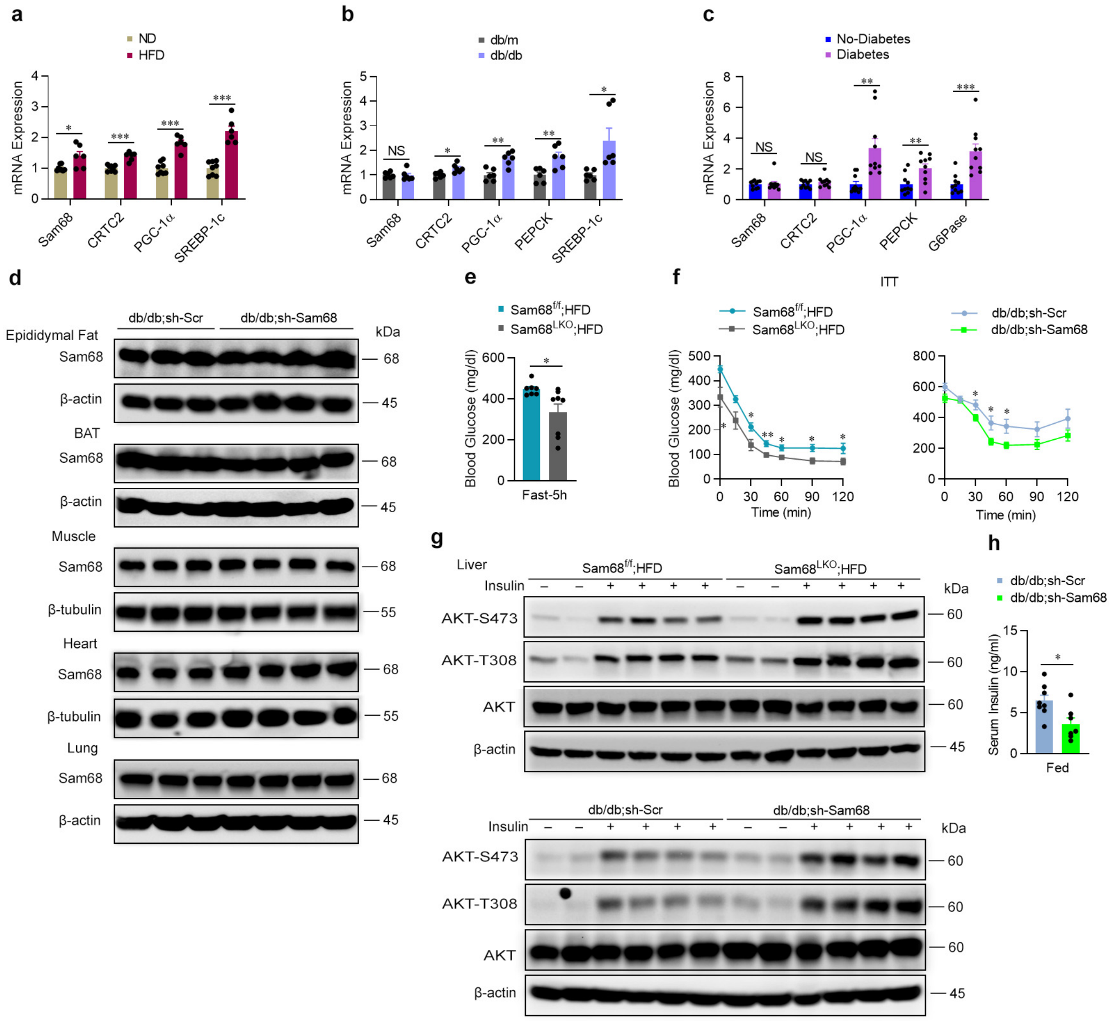
a
ND
HFD
mRNA Expression
Sam68
CRTC2
PGC-1α
SREBP-1c
b
db/m
db/db
c
No-Diabetes
Diabetes
PEPCK
G6Pase
d
db/db;sh-Scr
db/db;sh-Sam68
kDa
Epididymal Fat
BAT
Muscle
Heart
Lung
β-actin
β-tubulin
e
Sam68f/f;HFD
Sam68LKO;HFD
Blood Glucose (mg/dl)
Fast-5h
f
ITT
Time (min)
g
Liver
Insulin
AKT-S473
AKT-T308
AKT
h
Serum Insulin (ng/ml)
Fed

Supplementary Fig. 6: Hepatic Sam68 inactivation reduces gluconeogenic gene expression and blood-glucose levels in diabetic mice. (**a-b**) mRNA expression of *Sam68*, *CRTC2*, and *SREBP-1c*, and gluconeogenic genes was evaluated via qRT-PCR in the livers of (**a**) WT mice fed a normal diet (ND, n=8) or a HFD (n=6) for 3 months and (**b**) *db/db* (n=6) and control *db/m* (n=6) mice at age 2-3 months. (**c**) mRNA expression of *Sam68*, *CRTC2*, and gluconeogenic genes was measured in the livers of patients with or without diabetes (n=10 per group). (**d**) Sam68 protein levels were evaluated in the epididymal fat, brown fat, skeletal muscle, heart, and lung of *db/db*;sh-Scr and *db/db*;sh-Sam68 mice. (**e-f**) Blood glucose levels were measured in (**e**) $Sam68^{f/f}$;HFD (n=7) and $Sam68^{LKO}$;HFD (n=8) mice after 5 h of fasting and in (**f**) $Sam68^{f/f}$;HFD (n=7), $Sam68^{LKO}$;HFD (n=8), *db/db*;sh-Scr (n=8) and *db/db*;sh-Sam68 (n=8) mice during the insulin tolerance test (ITT). (**g**) Mice were treated with (+) or without (–) insulin (1 U/kg), and protein levels of phosphorylated AKT (at amino acids S473 and T308) and total AKT were evaluated 20 min later. (**h**) Serum insulin levels in *db/db*;sh-Scr (n=8) and *db/db*;sh-Sam68 (n=7) at feeding condition. Data are expressed as mean ± standard error of the mean (s.e.m.) *p<0.05, **p<0.01, ***p<0.001, NS, not significant (a-c, e, h: unpaired two-sided *t* test, f: two-way ANOVA). Source data are provided as a Source Data file.

Supplementary Tables

Supplementary Table 1: Demographic information of diabetic and non-diabetic patients

Age (years)	Sex	Ethnicity	Diabetes	Medications
48	Female	Caucasian	Yes	Metformin
36	Female	Caucasian	Yes	Metformin
56	Female	Caucasian	Yes	Metformin
78	Female	Caucasian	Yes	Metformin
68	Female	Caucasian	Yes	Metformin
74	Female	Asian	Yes	Metformin
54	Male	Caucasian	Yes	Insulin
58	Male	Caucasian	Yes	Insulin
73	Male	Caucasian	Yes	Insulin
71	Male	African American	Yes	Metformin
49	Female	Caucasian	No	N/A
41	Female	Caucasian	No	N/A
44	Female	Caucasian	No	N/A
31	Female	Caucasian	No	N/A
46	Female	African American	No	N/A
32	Female	Caucasian	No	N/A
52	Female	Caucasian	No	N/A
42	Female	African American	No	N/A
62	Female	Caucasian	No	N/A
50	Female	Caucasian	No	N/A

Supplementary Table 2: List of antibodies

Name	Catalogue Number	Company Name	Dilution
PGC-1α	ab54481	Abcam	1:1000
G6Pase	ab83690	Abcam	1:1000
Sam68 for WB	ab76472	Abcam	1:5000
PEPCK	12940	Cell Signaling	1:1000
FOXO1	2880	Cell Signaling	1:1000
β-actin	8457	Cell Signaling	1:1000
Lamin A/C	4777	Cell Signaling	1:1000
α-tubulin	2144	Cell Signaling	1:1000
β-tubulin	2146	Cell Signaling	1:1000
Myc-Tag	2276	Cell Signaling	1:1000
p-AKT (Thr308)	2965	Cell Signaling	1:1000
p-AKT (Ser473)	4060	Cell Signaling	1:1000
AKT	9272	Cell Signaling	1:1000
p-CREB (Ser133)	9196	Cell Signaling	1:1000
CREB	9197	Cell Signaling	1:1000
Phospho-(Ser/Thr) PKA substrate	9621	Cell Signaling	1:1000
LC3B	2775	Cell Signaling	1:1000
CRTC1	2587	Cell Signaling	1:1000
CRTC3	2720	Cell Signaling	1:1000
Anti-mouse IgG, HRP-linked antibody	7076	Cell Signaling	1:5000
Anti-rabbit IgG, HRP-linked antibody	7074	Cell Signaling	1:5000
HA-Tag	26183	Thermo Fisher	1:2000
CRTC2 for WB	PA5-72994	Thermo Fisher	1:1000
GcR	ABS551MI	Thermo Fisher	1:2000
HNF4α	MA1199	Thermo Fisher	1:1000
Flag-Tag	F3165	Sigma-Aldrich	1:5000
CRTC2 for IP	sc-271912	Santa Cruz	N/A
Normal mouse IgG-AC	sc-2343	Santa Cruz	N/A
Sam68 (7-1) for IP	sc-1238	Santa Cruz	N/A
Ubiquitin	VU101	LifeSensors	1:500
P62/SQSTM1	NBP1-48320	Novusbio	1:1000

Supplementary Table 3: List of primers

	Forward sequence	Reverse sequence
**For assessment of gene expression with qRT-PCR**		
mSREBP-1c	GGAGCCATGGATTGCACATT	GGCCCGGGAAGTCACTGT
mPGC-1α	AGCCGTGACCACTGACAACGAG	GCTGCATGGTTCTGAGTGCTAAG
mPEPCK	CCACAGCTGCTGCAGAACA	GAAGGGTCGCATGGCAAA
mG6pase	TGGGCAAAATGGCAAGGA	TCTGCCCCAGGAATCAAAAAT
mCRTC1	CATGATGGAGAACGCCATCAG	CACCGTGAGGATGATGTTGGG
mCRTC2	TTTGGGCATCAGTGGAGGTC	CCTGGAGGTTGGGATTGCTT
mCRTC3	CACAGTCAGACTTCCAGCTC	ATGGGTTTGGTCTCAAGTGG
mPkar1α	CCCTCGAGTCAGTACGGATG	GCATTCCTTCGGGAATACTTT
mPkar2α	CCTCCTCTTCTTCATCAGGG	GAGTGACTCGGACTCGGAAG
mPkar2β	GATCATCGGTTTTGGGATGT	ATAAACCGGTTCACAAGGCG
mPkacα	ATTCTGAGAAGGGGTCTCCC	AAGAAGGGCAGCGAGCAG
mPkacβ	TCCTCAAGCCCAGCATTACT	CAAGAAAGGCAGCGAAGTG
mGcR	ATTGGCGATGACCTCAGTGTGA	GCAATAGTTGGCTATGATGCCG
mβ-actin	GTATGGAATCCTGTGGCATC	AAGCACTTGCGGTGCACGAT
mSam68	GATATCTGTCAGGAGCAGTTTCT	CTCCTCGTCCTCTCACAGATA
hPGC-1α	AACAGCAGCAGAGACAAATGCACC	TGCAGTTCCAGAGAGTTCCACACT
hPEPCK	AAGGAGGATGCCCTGAACCTGAAA	TGCACCTTATGGATGGGAAAGGGA
hG6Pase	TGAATGGCTGCAGTGACCCAGATA	TGGATGTGGAGCCAGTGGAAGAAT
hCRTC2	CTCTGCCCAATGTTAACCAGAT	GAGTGCTCCGAGATGAATCC
hβ-actin	AGGATGCAGAAGGAGATCACTG	GGGTGTAACGCAACTAAGTCATAG
h-Sam68	GCGAGTGCTGATACCTGTCAAG	TCATTGAGCCCTTTCCCAAT
**For ChIP-qPCR**		
mPGC-1α-promoter	GGGCTGCCTTGGAGTGACGTC	AGTCCCCAGTCACATGACAAAG
mG6Pase-promoter	GGAGGGCAGCCTCTAGCACTGTCAA	TCAGTCTGTAGGTCAATCCAGCCCT
mPEPCK-promoter	GGCCTCCCAACATTCATTAAC	GTAGCCCGCCCTCCTTGCTTTA
**For generation of Sam68f/f mice**		
5' arm PCR	CCAAGGCCTCCTCATCTGATG	GTCTACACACAAAGCCCCGAG
Central fragment	CTGCCCGGCTTCTTGAGTAAG	TCCCCTACTTGTCGGCTCTAC
3' arm PCR	TAGCACCAAGCTCCCTCCAAG	TCCTGTTCCCAACGTCACCAG
ES screening PCR	GATTCGCAGCGCATCGCCTTCT	TACCGGTGGATGTGGAATGTG
5' arm PCR Dig	CAGGGTTTCTCTGTGTAGCCC	AGTGGCGCACGCCTTTAATCC
F or W genotyping	TTGGGAAAGAGGTATGGCTTGGCA	AAGAAGTTCCTGCCTAACTCTCCC
**For genotyping of Sam68^{LKO} mice**		
Alb-Cre	TGCCTGCATTACCGGTCGATGC	CCATGAGTGAACGAACCTGGTCG
**For genotyping of Sam68$^{\Delta N\text{-}Tg}$ mice**		
Exon6F-Exon7R	TAGAGGAGCTTTGGTTCGTG	AATAGCCTTCATAGCCTTCG
