# Supplementary material for: Sam68 promotes hepatic gluconeogenesis via CRTC2
Source: Nat Commun. 2021 Jun 7;12:3340. doi: 10.1038/s41467-021-23624-9 (PMC8185084; doi:10.1038/s41467-021-23624-9)
Supplement: Supplementary file 3 — Reporting Summary [file 41467_2021_23624_MOESM3_ESM.pdf]

nature research

Corresponding author(s): Gangjian Qin

Last updated by author(s): Apr 10, 2021

Reporting Summary

Nature Research wishes to improve the reproducibility of the work that we publish. This form provides structure for consistency and transparency in reporting. For further information on Nature Research policies, see our Editorial Policies and the Editorial Policy Checklist.

Statistics

For all statistical analyses, confirm that the following items are present in the figure legend, table legend, main text, or Methods section.

n/a	Confirmed	
☐	☒	The exact sample size (*n*) for each experimental group/condition, given as a discrete number and unit of measurement
☒	☐	A statement on whether measurements were taken from distinct samples or whether the same sample was measured repeatedly
☐	☒	The statistical test(s) used AND whether they are one- or two-sided<br>*Only common tests should be described solely by name; describe more complex techniques in the Methods section.*
☐	☒	A description of all covariates tested
☐	☒	A description of any assumptions or corrections, such as tests of normality and adjustment for multiple comparisons
☐	☒	A full description of the statistical parameters including central tendency (e.g. means) or other basic estimates (e.g. regression coefficient) AND variation (e.g. standard deviation) or associated estimates of uncertainty (e.g. confidence intervals)
☐	☒	For null hypothesis testing, the test statistic (e.g. *F*, *t*, *r*) with confidence intervals, effect sizes, degrees of freedom and *P* value noted<br>*Give P values as exact values whenever suitable.*
☒	☐	For Bayesian analysis, information on the choice of priors and Markov chain Monte Carlo settings
☒	☐	For hierarchical and complex designs, identification of the appropriate level for tests and full reporting of outcomes
☒	☐	Estimates of effect sizes (e.g. Cohen's *d*, Pearson's *r*), indicating how they were calculated

Our web collection on statistics for biologists contains articles on many of the points above.

Software and code

Policy information about availability of computer code

Data collection	Western blotting data were collected on Bio-Rad ChemiDoc System. The PCR data were collected with ABI3000 Sequence Detector system (Applied Biosystems). Blood glucose levels were measured with a Bayer Contour Blood Glucose Meter. Hyperinsulinemic-euglycemic clamping was performed on syringe pumps (CMA 402, Harvard Apparatus, Holiston, MA; NE-300, New Era Pump Systems, Farmingdale, NY). Radioactivities were measured with a Multi-purpose Scintillation Counter LS6500.
Data analysis	Prism8 Software was used for statistical analysis. Western blotting data were analyzed with NIH Image J bundled with 64-bit Java 1.8.0.

For manuscripts utilizing custom algorithms or software that are central to the research but not yet described in published literature, software must be made available to editors and reviewers. We strongly encourage code deposition in a community repository (e.g. GitHub). See the Nature Research guidelines for submitting code & software for further information.

Data

Policy information about availability of data

All manuscripts must include a data availability statement. This statement should provide the following information, where applicable:

- Accession codes, unique identifiers, or web links for publicly available datasets
- A list of figures that have associated raw data
- A description of any restrictions on data availability

All data are included in the Manuscript and Supplementary Information files, or available from the authors upon reasonable request.

Field-specific reporting

Please select the one below that is the best fit for your research. If you are not sure, read the appropriate sections before making your selection.

☒ Life sciences ☐ Behavioural & social sciences ☐ Ecological, evolutionary & environmental sciences

For a reference copy of the document with all sections, see nature.com/documents/nr-reporting-summary-flat.pdf

Life sciences study design

All studies must disclose on these points even when the disclosure is negative.

Sample size	Sample sizes were chosen based on our previous similar studies with mouse blood glucose measurements, euglycemia-hyperinsulinemia clamping, and diabetic models (Qiao et al, JCB 2017;216:723-741. Zhou J et al, J Endocrinol 2015;225:181-9. Kim T et al, Diabetes 2018;67:2157-2166) and consistent with the recommended standard operating procedures for performing metabolic tests of glucose in mice (Julio E. Ayala et al, Dis Model Mech 2010;3:525-34).
Data exclusions	No completed data were excluded from the experiments presented in this manuscript.
Replication	Animal experiments were performed with sufficient sample sizes as stated above. All primary cell experiments were performed with n=3-4 biologically independent cell samples per group and repeated at least twice. All representative data were repeated at least twice unless stated otherwise.
Randomization	Age and sex matched mice were randomized to receive different treatments, including Sam68f/f mice to receive AAV8-TBG-eGFP or AAV8-TBG-iCre virus, Sam68f/f and Sam68LKO mice to receive Ad-GFP or Ad-CRTC2K628R virus, and ob/ob mice to receive AAV8-GFP-murinSam68-shRNA or AAV8-GFP-Scrmb-shRNA virus. The liver samples from human subjects were not additionally selected for biochemical analyses.
Blinding	The investigators were blinded to group allocation during data collection and analysis.

Reporting for specific materials, systems and methods

We require information from authors about some types of materials, experimental systems and methods used in many studies. Here, indicate whether each material, system or method listed is relevant to your study. If you are not sure if a list item applies to your research, read the appropriate section before selecting a response.

Materials & experimental systems

n/a	Involved in the study	
☐	☒	Antibodies
☐	☒	Eukaryotic cell lines
☒	☐	Palaeontology and archaeology
☐	☒	Animals and other organisms
☐	☒	Human research participants
☒	☐	Clinical data
☒	☐	Dual use research of concern

Methods

n/a	Involved in the study	
☒	☐	ChIP-seq
☒	☐	Flow cytometry
☒	☐	MRI-based neuroimaging

Antibodies

Antibodies used	Western blotting analyses were performed by using the antibodies for PGC-1α (Abcam, ab54481, 1:1000), G6Pase (Abcam, ab83690, 1:1000), Sam68 (Abcam, ab76472,1:5000), PEPCK (Cell Signaling, 12940, 1:1000), β-actin (Cell Signaling, 8457, 1:000), Lamin A/C (Cell Signaling, 4777,1:1000), β-tubulin (Cell Signaling, 2146, 1:1000), α-tubulin (Cell Signaling, 2144, 1:1000), Myc-Tag (Cell Signaling, 2276, 1:1000), p-AKT (Thr308) (Cell Signaling, 2965,1:1000), p-AKT (Ser473) (Cell Signaling, 4060, 1:1000), AKT (Cell Signaling, 9272, 1:1000), p-CREB (Ser133) (Cell Signaling, 9196, 1:1000), CREB (Cell Signaling, 9197, 1:1000), FOXO1 (C29H4) (Cell Signaling, 2880, 1:1000), Phospho-(Ser/Thr) PKA substrate (Cell Signaling, 9621, 1:1000), LC3B (Cell Signaling, 2775, 1:1000), CRTC1 (Cell Signaling, 2587, 1:1000), CRTC3 (Cell Signaling, 2720, 1:1000), HA-Tag (Thermo Fisher, 26183, 1:2000), CRTC2 (Thermo Fisher, PA5-72994, 1:1000), GcR (Thermo Fisher, ABS551MI, 1:2000), HNF4α (Thermo Fisher, MA1199, 1:1000), Flag-Tag (Sigma-Aldrich, F3165, 1:500), Ubiquitin (LifeSensors, VU101, 1:500), P62/SQSTM1 (Novusbio, NBP1-48320, 1:1000), Anti-mouse IgG, HRP-linked antibody (Cell Signaling, 7076, 1:5000), Anti-rabbit IgG, HRP-linked antibody (Cell Signaling, 7074, 1:5000).<br>Co-immunoprecipitation experiments were performed by using anti-CRTC2 (Santa Cruz, sc-271912), anti-Sam68 (Santa Cruz, sc-1238), anti-mouse IgG-AC (Santa Cruz, sc-2343), anti-Flag (Sigma-Aldrich, F3165), and anti-HA (Thermo Fisher, 26183).<br>Chromatin immunoprecipitation experiments were performed by using anti-CRTC2 (Santa Cruz,sc-271912).
Validation	All primary antibodies used in this report were obtained from commercial sources. The validation information can be found online for antibodies from Cell Signaling (https://www.cellsignal.com/about-us/cst-antibody-validation-principles), Thermo Fisher (https://www.thermofisher.com/us/en/home/life-science/antibodies/invitrogen-antibody-validation.html?icid=ab-search-learning-ab-validation), and Abcam (https://www.abcam.com/primary-antibodies/how-we-validate-our-antibodies). The anti-Ubiquitin from

LifeSensors has been validated (https://lifesensors.com/wp-content/uploads/2019/08/VU101_VU-1Antibody_datasheet_032113.pdf) and used extensively in the field (Wilkinson KD et al, Faseb J 1997;11:1245-56. Meulmeester E et al, Mol Cell 2005;18:565-76. Li et al, Nature 2002;416:648-53. Seiberlich et al, Biochem et Biophys Acta 2012;1823:2057-2068). The anti-P62/SQSTM1 from Novusbio has been validated (https://www.novusbio.com/PDFs/NBP1-48320.pdf). The anti-CRTC2 from Santa Cruz has been extensively by many other labs (Seok S et al, Nature 2014;516: 108-111. Woo J and Kang S, Lipids Health Dis 2016;15:147. Chen X et al, FASEB J 2020 ePub). The anti-Sam68 from Santa Cruz has been used by many other labs, and we validated this antibody by Western blotting analysis in WT vs. Sam68 knockout mice as reported in our previous reports (Zhou J et al, J Endocrinol 2015;225:181-9. Han S et al, J Mol Cell Cardiol 2019;137:82-92) and in this article.

Eukaryotic cell lines

Policy information about cell lines

Cell line source(s)	HepG2 cells and HEK293T cells were purchased from the American Type Culture Collection (ATCC, Rockville, MD, USA).
Authentication	All cell lines have been authenticated by STR profiling (ATCC). The primary cell lines were not further authenticated.
Mycoplasma contamination	Our lab has routine testing to make sure no mycoplasma contamination in the cell culture system. The primary cell lines used in this study were not specifically tested for mycoplasma contamination.
Commonly misidentified lines (See ICLAC register)	No cell line used in our study is listed in the ICLAC register.

Animals and other organisms

Policy information about studies involving animals; ARRIVE guidelines recommended for reporting animal research

Laboratory animals	Experiments were conducted in 2 to 3 month-old male mice on C57BL/6j background unless specified otherwise. Sam68flox/flox mice and Sam68ΔN transgenic mice were generated in our lab as detailed in this article. Alb-Cre+;Sam68f/f (Sam68LKO) mice and Sam68f/f littermates were produced by crossing Sam68f/f mice with Alb-cre mice (The Jackson Laboratory, No. 003574). Sam68+/– mice were generated by Dr. Stéphane Richard at McGill University, Montreal, Canada (Richard S et al. 2005) and bred to obtain Sam68–/– (Sam68-KO) mice and WT littermates. db/db and db/m mice were purchased from Jackson Laboratory (No. 000642). Mice were fed ad libitum and maintained in a climate-controlled facility (22°C, 43% humidity) with a 12:12-h light:dark cycle.
Wild animals	No wild animals were used.
Field-collected samples	No field-collected samples were used.
Ethics oversight	All animal experiments in this report were approved by the Institutional Animal Care and Use Committees (IACUC) of Northwestern University and the University of Alabama at Birmingham and comply with relevant ethical regulations, including the National Institutes of Health (NIH) ‘‘Guide for the Care and Use of Laboratory Animals’’.

Note that full information on the approval of the study protocol must also be provided in the manuscript.

Human research participants

Policy information about studies involving human research participants

Population characteristics	Diabetic and non-diabetic patients' characteristics are listed in Supplementary Table 1. One group contains 10 diabetic patients (Age: 36-71 years old; Sex: 4 male and 6 female; Ethnicity: 8 white, 1 black, and 1 asian; Liver Disease: 3 hepatic adenoma [benign], 2 normal, 5 N/A; Medications: 7 on metformin, 3 on insulin). The other group contains 10 non-diabetic patients (Age: 31-62 years old; Sex: all females; Ethnicity: 8 white, 2 black; Liver Disease: 7 hepatic adenoma [benign], 2 normal, 1 hemangioma [Benign]; Medications: N/A).
Recruitment	The liver samples were obtained via surgical biopsy from patients with or without diabetes. Informed consents were obtained from all subjects, and there was no selection bias in the sample analyses.
Ethics oversight	Studies with human tissues were approved by the Institutional Review Board (IRB) for Human Use of the University of Alabama at Birmingham (Protocol #: IRB-300002079) and performed in compliance with the Belmont Report and Declaration of Helsinki.

Note that full information on the approval of the study protocol must also be provided in the manuscript.
